# Exploring the Evolvability of Plant Specialized Metabolism: Uniqueness Out Of Uniformity and Uniqueness Behind Uniformity

**DOI:** 10.1093/pcp/pcad057

**Published:** 2023-06-12

**Authors:** Eiichiro Ono, Jun Murata

**Affiliations:** Suntory Global Innovation Center Ltd. (SIC), 8-1-1 Seikadai, Seika-cho, Soraku-gun, Kyoto, 619-0284 Japan; Bioorganic Research Institute (SUNBOR), Suntory Foundation for Life Sciences, 8-1-1 Seikadai, Seika-cho, Soraku-gun, Kyoto, 619-0284 Japan

**Keywords:** Metabolic convergence, Metabolic divergence, Plant specialized metabolism

## Abstract

The huge structural diversity exhibited by plant specialized metabolites has primarily been considered to result from the catalytic specificity of their biosynthetic enzymes. Accordingly, enzyme gene multiplication and functional differentiation through spontaneous mutations have been established as the molecular mechanisms that drive metabolic evolution. Nevertheless, how plants have assembled and maintained such metabolic enzyme genes and the typical clusters that are observed in plant genomes, as well as why identical specialized metabolites often exist in phylogenetically remote lineages, is currently only poorly explained by a concept known as convergent evolution. Here, we compile recent knowledge on the co-presence of metabolic modules that are common in the plant kingdom but have evolved under specific historical and contextual constraints defined by the physicochemical properties of each plant specialized metabolite and the genetic presets of the biosynthetic genes. Furthermore, we discuss a common manner to generate uncommon metabolites (uniqueness out of uniformity) and an uncommon manner to generate common metabolites (uniqueness behind uniformity). This review describes the emerging aspects of the evolvability of plant specialized metabolism that underlie the vast structural diversity of plant specialized metabolites in nature.

## Introduction: From the Origin

Plant specialized metabolism, also called plant secondary metabolism, is a generic term for the biochemical mechanism that biosynthesizes an array of metabolites; many of these metabolites are valuable for the environmental interaction of land plants with other organisms (Arimura et al. 2009), while some other metabolites, in the form of medicines or spices, are beneficial to humans. The biosynthetic pathways of plant specialized metabolites are characterized by their biosynthetic origins that typically branch out from the biosynthesis of primary core metabolic pathways for amino acids, nucleic acids and lipids that are highly conserved across the plant kingdom (Weng 2014, Weng et al. 2021). In contrast to the apparent commonalities in the chemical structures and the occurrence of such biosynthetic precursors across various plant species, plant specialized metabolites sharing core structures usually occur in a lineage-specific manner. However, these metabolites exhibit vast diversity in their overall structures and biological activities (**Table 1**) (de Vries et al. 2021), both of which have likely contributed to increased hidden ecological fitness in diverging and fluctuating environments.

**Fig. 1:**
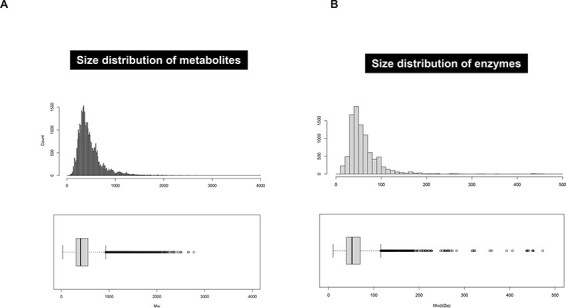
The size distribution of metabolites and enzymes. (A) The size distribution of plant metabolites calculated from 61,520 metabolites listed in the KNApSAcK database (http://www.knapsackfamily.com/KNApSAcK_Family/) (Afendi et al. 2012). The median size of metabolites is 402.2 with a 95% confidence interval (CI): 400.4 < median < 403.9 (minimum: 27.0, 1/4: 308.0, 3/4: 556.1 and maximum: 928.2). (B) The size distribution of 9,006 enzymes (i.e. proteins with catalytic sites) from *A. thaliana, Nicotiana tabacum* and *Sesamum indicum* deposited in the UniProt Knowledgebase (https://www.uniprot.org/uniprotkb?query=*). The median size of the enzymes is calculated to be 51.8 kDa with a 95% CI: 51.3 < median < 52.3 (minimum: 10.1, 1/4: 39.6, 3/4: 69.8 and maximum: 115.2) using the following formula: protein molecular weight (kDa) = amino acid length (a.a.) × 120 (average amino acid molecular weight). The upper and lower graphs indicate a histogram and box plot for each distribution, respectively.

Most plant specialized metabolites are composed of low-molecular-weight organic compounds (∼1,000 *m/z*) and are likely associated with enzymes, transporters and other biosynthetic machineries, which are typically composed of polypeptides (∼100 kDa). Size distributions of metabolites in the KNApSAcK database and proteins with catalytic sites in the UniProt Knowledgebase have a median molecular weight of 402.2 *m/z* and 51.8 kDa, respectively (Afendi et al. 2012) (**Fig. 1**). The notable similarity in the two molecular size distribution curves suggests that the metabolite sizes are restricted by the enzyme sizes.

The structural uniqueness of specialized metabolites suggests that their evolutionary origins are relatively recent compared with conserved and common metabolites such as primary metabolites and plant hormones. In general, specialized metabolites are more abundant than phytohormones that share common precursors (**Table 1**). For example, both glucobrassicin, a specialized indole glucosinolate, and indole acetic acid, a phytohormone auxin, are commonly derived from tryptophan. However, their relative abundances differ by approximately 2,000-fold in the aerial parts of 2-week-old seedlings of *Arabidopsis thaliana* (Sugahara et al. 2020). Moreover, the levels of specialized steviol glycosides and the plant hormone gibberellins, both of which are geranylgeranyl pyrophosphate–derived structurally related diterpenoids, are estimated to differ in abundance by around 10,000-fold in the leaves of *Stevia rebaudiana* (Brahmachari et al. 2011). These significant differences in the abundances of phytohormones and specialized metabolites raise an intriguing question regarding whether there is a correlation between the quantity of metabolites and the elapsed time since metabolic evolution because the amounts of metabolites required to perform given biological functions would have been optimized in terms of cellular cost. This is analogous to catalytic optimization in which the high expression of a low-activity enzyme is replaced by the low expression of a highly active enzyme. Whether low metabolic flux levels emerged as such from the beginning of the metabolic pathway or whether highly active metabolic pathways have evolved suddenly remain an open question. In all likelihood, both the activity and quantity of enzymes would have been continuously optimized during metabolic evolution with respect to cellular cost.

**Table 1:** Summary of contrasting properties between core and specialized metabolisms

	Core metabolites	Specialized emtabolites
Distribution	Common for absolutely conserved	Often lineage specific and sometimes sporadic
Evolutional origin	Ancient	Recent
Metabolic position	Central	Peripheral at the frontier of biological interaction
Chemical structures	Universal	Diverse, but with common reactions
Flux	Generally optimized	Locally accumulative
Evolvability	Constraint by numerous entanglements	Accelerated by multiple presets of common units

On the other hand, low levels of metabolites might also reflect their biochemical nature, either as pathway intermediates that are rapidly metabolized by highly catalytic enzymes or as toxic and/or highly reactive compounds that are too detrimental to be stably accumulated in plants. The latter scenario is exemplified by an adaptation to the dilemma of evolving rattlesnake venom toxins and avoiding self-poisoning by tailoring proteins called auto-inhibitors (Ukken et al. 2022). Plants have successfully avoided self-intoxication by toxic metabolites (autotoxicity) via conjugation (e.g. glycosylation, methylation and acylation), sequestration to specialized organs or intracellular organelles or secretion into extracellular spaces. Each of these methods enables the stable accumulation of specialized metabolites and avoids autotoxicity. Other than detoxification, plants can adapt to highly toxic metabolites. For example, the adaptive evolution of topoisomerase I with tolerant mutations against the specialized alkaloid camptothecin, which exerts anticancer effects via the inhibition of topoisomerase I, is an example of an alternative strategy for avoiding the problem of autotoxicity (Sirikantaramas et al. 2008).

The biological relevance of plant specialized metabolism has often been ascribed to enhancing ecological fitness against various biotic and abiotic stresses, rather than to the autonomous control of cell and tissue development of the plants (Huang et al. 2019). This is partly because it has generally been challenging to relate the developmental control of primary metabolism, plant hormones and other biological mechanisms that are highly conserved in the plant kingdom with plant specialized metabolites that are found only in selected plant species.

However, increasing evidence reveals that, in some instances, specialized metabolism can exert control of plant tissue development. For example, the enzymatic activity of thalianol synthase and thalianol acyltransferase 2, which are involved in the biosynthesis of thalianol and other triterpenes in *A. thaliana*, has been shown to control root development (Bai et al. 2021). The sequential emergence of plant hormones during evolution already suggests that plant hormones are conserved in plants as only an evolutional consequence of the selection pressures for their possession in a common ancestral plant and that small molecules that are currently categorized as ‘specialized metabolites’ are eligible to become new plant hormones in the future. In this context, the births of lineage-specific metabolic branches and extensions from central metabolism, which have repetitively and frequently occurred during evolution, have been the frontier of metabolic evolution. This in turn suggests that the current ‘border’ between primary and specialized metabolism is defined merely by a tentative snapshot during metabolic evolution that is more dynamic than expected (**Table 1**).

Genomic approaches have accelerated the complete identification of the whole set of biosynthetic enzymes involved in various classes of plant specialized metabolites and have revealed that these enzyme genes can be grouped into a couple of classes despite the diversity in the structures of plant specialized metabolites (Lau and Sattely 2015, Qu et al. 2019, Hong et al. 2022, La Peña R et al. 2023). More specifically, specialized metabolism is a flow of enzyme-catalyzed structural changes in substrate molecules through oxidation, reduction and conjugation modification (glycosylation, methylation, acylation, etc.), where cytochrome P450 monooxygenases (CYP), 2-oxoglutarate-dependent dioxygenases (2ODD/DOX), UDP sugar-dependent glycosyltransferases (UGTs), *S*-adenosylmethionine-dependent *O*-methyltransferases (OMTs) and acyltransferases are universally involved as the common catalytic units (Kawai et al. 2014, Wilson and Tian 2019, Hansen et al. 2021). Thus, it is likely that gene multiplication and the neo-functionalization of a limited set of family enzymes have acted together to generate specialized metabolites for structural uniqueness.

These enzyme genes constitute superfamilies with dozens to hundreds of structurally related genes in a given plant genome. Although there is still much to debate around the putative molecular mechanisms of how such superfamilies of enzyme genes evolved, the combination of whole-genome duplication, which multiplies all the genes in a genome, and local tandem duplication, which multiplies a selected region in the genome, are likely to be critical for the lineage-specific specialization of metabolic pathways (Chae et al. 2014, Zhan et al. 2022). Enzyme genes in a given superfamily usually have different catalytic specificities owing to differences in spatiotemporal expression and amino acid sequences responsible for the catalytic specificity. The high copy number variations of enzyme genes suggest not only the biochemical diversity and the resilience of metabolism but also the versatility of these superfamily genes based on their plasticity and multiplicity. In this review, we focus on new aspects of metabolic evolvability, which enable an increase in the structural diversity of specialized metabolites in nature.

## Metabolic Convergence in Phylogenetically Distant Plants

Oxidation and glycosylation reactions frequently occur sequentially in plant hormone metabolism and specialized metabolism, thereby increasing the structural diversity and the water solubility of metabolites in plant cells (Kawai et al. 2014). For example, it has been revealed that the specialized glycoalkaloid metabolism of tomatoes and potatoes in the Solanaceae family diverged from a common triterpene pathway by recruiting different enzymes (Akiyama et al. 2021b, 2022). This is a typical example of lineage-specific specialized metabolism formed by lineage-specific enzyme evolution, also known as divergent evolution, which forms a chemotaxonomic group sharing similar specialized metabolites.

Although specialized metabolites essentially have lineage-specific genetic origins, therefore observed among only taxon-restricted phylogenetically close species, they are often sporadically observed in phylogenetically distant lineages. The discontinuous presence of identical specialized metabolites has essentially been recognized to result from the convergent evolution of metabolic pathways through the independent development of biosynthetic enzymes in phylogenetically unrelated lineages. Recent metabolomic analysis revealed that the convergent evolution of specialized metabolism is not uncommon; examples include the biosynthesis of flavonoids (aurone, flavone, etc.) (Martens and Mithöfer 2005, Davies et al. 2020), cyanogenic glycosides (Takos et al. 2011), coumarins (Munakata et al. 2021, Villard et al. 2021), alkaloids (e.g. caffeine, nesocodin and various benzylisoquinoline alkaloids) (Huang et al. 2016, Li et al. 2020, O’donnell et al. 2021, Roy et al. 2022), acylsugars (Lou et al. 2021), diterpenes (e.g. momilactone) (Mao et al. 2020), triterpenes (e.g. diosgenin) (Christ et al. 2019) and cannabinoids (Berman et al. 2023). Remarkably, lignans (e.g. podophyllotoxin), cyanogenic glucosides and diterpene-derived phytohormones are known to undergo convergent evolution in insects and fungi beyond plants (Beran et al. 2019, Hirai et al. 2000, Tudzynski 2005, Jensen et al. 2011, Boyce et al. 2019, Tran et al. 2022) (**Table 2**).

**Table 2:** Examples of sporadically distributed common specialized metabolites in plants

		Genus (family)		
Class	Specialized metabolite^a^	Plant 1	Plant 2	MYA^b^
Lignan	Sesamin	*Sesamum* (Pedaliaceae)	*Paulownia* (Paulowniaceae)	42
			*Artemisia* (Asteraceae)	101
			*Zanthoxylum* (Rutaceae)	120
			*Asarum* (Aristolochiaceae)	160
			*Piper* (Piperaceae)	160
			*Magnolia* (Magnoliaceae)	160
			*Ginkgo* (Ginkgoaceae)	330
	Deoxypodophyllotoxin	*Anthriscus* (Apiaceae)	*Podophyllum* (Berberidaceae)	129
			*Thujopsis* (Cupressaceae)	330
Diterpenoid	Momilactone	*Oryza* (Poaceae)	*Hypnum* (Hypnaceae)	488
	Stevioside	*Stevia* (Asteraceae)	*Rubus* (Rosaceae)	120
Triterpennoid	Diosgenin	*Dioscorea* (Dioscoreaceae)	*Trigonella* (Fabaceae)	160
Flavonoid (aurone)	Aureusidin/sulfuretin/auronidin	*Antirrhinum* (Plantaginaceae)	*Bidens* (Asteraceae)	101
			*Marchantia* (Marchantiaceae)	480
Courmarin	Bergamottin/isoimperatorin	*Citrus* (Rutaceae)	*Angelica* (Apiaceae)	120
Cyanogenic glucoside	Dhurrin/amygdalin/prunasin/lotaustrali/linamarin	*Lotus* (Fabaceae)	*Prunus* (Rosaceae)	99
			*Eucalyptus* (Myrtaceae)	108
			*Sorghum* (Poaceae)	160
			*Manihot* (Euphorbiaceae)	101
Purine alkaloid	Caffeine	*Camellia* (Theaceae)	*Coffea* (Rubiaceae)	108
			*Theobroma* (Malvaceae)	120
			*Citrus* (Rutaceae)	120

a Searched by KNApSAcK database (Afendi et al. 2012).

b Estimated divergent time (Million Years Ago) between two plants (1 and 2) by TimeTree (Kumar et al. 2022).

Possible molecular mechanisms behind the convergent evolution of identical specialized metabolites in phylogenetically distant plants include the gene repertories involved, enzymatic plasticity through gain or loss of genes, functional differentiation by spontaneous mutation and gene recombination under the physicochemical constraints of organic compounds, and biochemical constraints of catalysis.

## Catalytic Convergence in Enzymes

Sesamin, a specialized lignan that accumulates highly in dietary sesame seeds (*Sesamum* spp.), is observed not only in phylogenetically related species of Lamiales such as *Paulownia* spp. but also in phylogenetically distant *Cuscuta* of Solanales, *Zanthoxylum* spp. of Sapindales, *Houttuynia* spp. and *Piper* spp. of Piperales, *Magnolia* spp. of Magnoliales in the angiosperm and *Ginkgo* in the gymnosperm (Afendi et al. 2012, Kumar et al. 2022) (**Fig. 2**, **Table 2**). Although the physio-ecological functions of sesamin in plants are mostly unknown, this widespread sporadic presence of sesamin suggests untapped usefulness in increasing the fitness of the producing plants. Notably, CYP81Q1 from *Sesamum* spp. and its related gene from Lamiales are the only examples of a sesamin biosynthetic enzyme that catalyzes two sequential methylenedioxy bridge formations for the substrate pinoresinol, which is derived from two coniferyl alcohol molecules (Ono et al. 2006, Noguchi et al. 2014). The apparent lack of CYP81Q-related genes in sesamin-producing plant species other than Lamiales suggests that non-CYP81Q sesamin biosynthetic genes have evolved independently in other sesamin-producing plants by convergent metabolic evolution (Nelson and Werck-Reichhart 2011). The frequent occurrence of sesamin in unrelated lineages might reflect the fact that only relatively small numbers of catalytic steps would be required for sesamin biosynthesis from phenylpropanoid derivatives, such as coniferyl alcohol, which are highly conserved throughout the plant kingdom. This is also the case with caffeine, a specialized purine alkaloid sporadically found in many phylogenetically distant plant lineages (Huang et al. 2016) that does not require a long biosynthetic pathway.

**Fig. 2:**
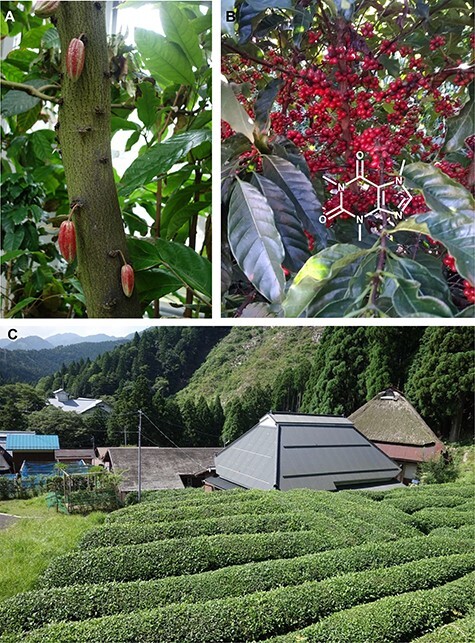
Examples of the sporadic distribution of convergent specialized metabolites in various plants. Caffeine is accumulated in (A) *Theobroma cacao* (Kyoto prefectural botanical garden, Japan), (B) *Coffea arabica* (Minas Gerais state, Brazil), and (C) *Camellia sinensis* (Mandokoro, Shiga

**Fig. 2:**
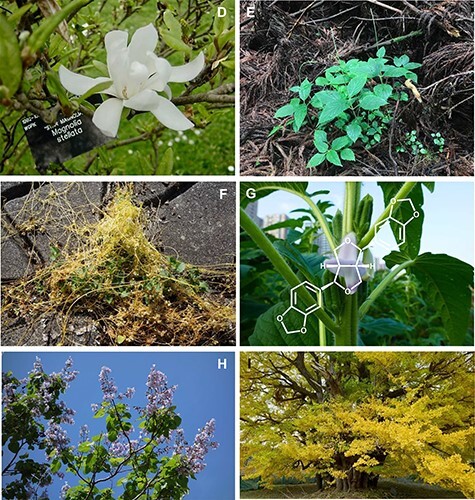
(Continued) prefecture, Japan). Sesamin-related lignans with methylenedioxy bridges have been found in (D) Flowers of *Magnolia stellate* (the Kew garden, London, UK), (E) *Phryma leptostachya* (Otowa mountain, Shiga prefecture), (F) *Cuscuta campestris* (parasiting on *Calystegia soldanella* landlocked on the lakeside of lake Biwa, Moriyama, Shiga prefecture, Japan), (G) A sesame cultivar, *Sesamum indicum* (Wuhan, China), (H) *Paulownia tomentosa* (Hanamaki, Iwate prefecture, Japan), and (I) *Ginkgo biloba* which is known as the amazing ‘big yellow’ and is estimated to be 1,000 years old (Kitakanegasawa, Aomori prefecture, Japan). Podophyllotoxin-related lignans with a methylenedioxy bridge are accumulated in (J) *Anthriscus sylvestris* (Ashiu Forest Research Station of Kyoto University, Japan), (K) *Thujopsis dolabrata* and (L) *Podophyllum peltatum* (Awaji botanical garden, Hyogo prefecture, Japan). Aurone-related flavonoids are found in (M) Yellow aurone flowers of *Antirrhinum majus* spatially regulated by a small RNA locus, *SULF* (Bradley et al. 2017), (N) *Linaria japonica* (Arid Land Research Center of Tottori University, Japan), (O) Yellow Campus, a yellow cosmos cultivar of *Cosmos bipinnatus* (Tamagawa University, Tokyo, Japan), and (P) *Marchantia polymorpha* grown in a laboratory.

**Fig. 2:**
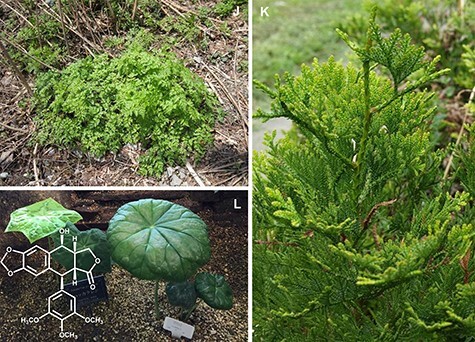
(Continued)

**Fig. 2:**
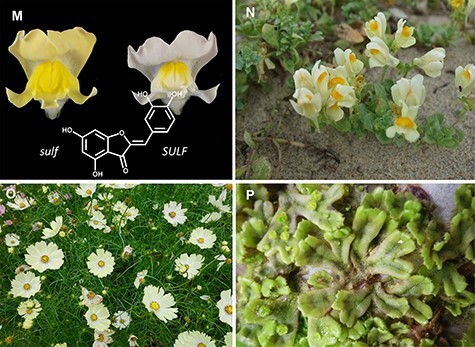
(Continued)

However, it is surprising that the biosynthetic enzymes of an antitumor lignan, (deoxy)podophyllotoxin, were recently identified in phylogenetically distant plants as its biosynthesis requires much greater complexity and many more reaction steps than sesamin and caffeine biosynthesis, even though it has been acquired in completely different lineages: cow parsley (*Anthriscus sylvestris* of Apiales), hinoki-asunaro (*Thujopsis dolabrata* var. hondae of Coniferae) and mayapple (*Podophyllum peltatum* of Ranunculales) (**Fig. 2**, **Table 2**). From these plants, there are three distinct OMTs that have been identified to catalyze methylation at the same position of their substrate lignans (Lau and Sattely 2015, Yamamura et al. 2023). Importantly, corresponding OMTs from those three different plant species share common amino acid substitutions in their substrate pockets. The data suggest that these OMTs were convergently incorporated into the specialized lignan metabolism under the functional constraint of recognizing the common substrate structure with common specificity. The common structural basis of the independently evolved metabolic enzymes with common specificity is known not only for the regiospecificity of OMTs but also for the regio-promiscuity of CYPs and the sugar-donor selectivity of UGTs (Ohgami et al. 2015, Christ et al. 2019, Ono et al. 2020). These studies highlight the necessity of functional plasticity and the genetic multiplicity of the enzyme for driving convergent metabolic evolution.

A well-known example of the convergent evolution of specialized flavonoids occurs in the biosynthesis of flavones. While the oxidation of flavanone to flavone is catalyzed by CYP in general, a DOX enzyme, flavone synthetase I, catalyzes the corresponding reaction in the Apiaceae family (Martens and Mithöfer 2005). Other notable examples of convergent evolution in specialized flavonoids are aurone synthases in the *Coreopsis* of Asteraceae, *Antirrhinum* (snapdragons) of Plantaginaceae and the liverwort *Marchantia* (Nakayama et al. 2000, Molitor et al. 2016, Berland et al. 2019, Davies et al. 2020, Furudate et al. 2023) (**Fig. 2**, **Table 2**). All aurone synthases are classified as polyphenol oxidases (PPOs), but their molecular lineages, substrate specificity and subcellular localization are different, revealing the parallel evolution of aurone biosynthesis-based functional plasticity of PPO enzymes (**Supplementary Fig. S1**).

The biosynthesis of cyanogenic glucosides is a thought-provoking example in considering specialized metabolic evolution. Their biosynthetic pathways in *Sorghum* (a grass), *Lotus* (a legume), *Manihot* (cassava) and *Eucalyptus* (of the Myrtaceae family) are commonly catalyzed by CYP79, CYP71 and UGT85 paralogs despite their phylogenetic distance (Kannangara et al. 2011, Takos et al. 2011, Thodberg et al. 2018, Hansen et al. 2022) (**Table 2**). Importantly, these paralogs are involved in the biosynthesis of glucosinolates, which are Brassicaceae-specialized metabolites known as ‘mustard oil bomb’. In the parallel evolution scenario, these structurally similar enzymes are only paralogous, not orthologous, and have been independently recruited from the same enzyme family. The exceptional reactions specifically catalyzed by CYP736 in *Lotus* and CYP706 and UGT87 in *Eucalyptus*, respectively, which are apparently lineage-specific recruited enzymes (Takos et al. 2011, Hansen et al. 2022), support the parallel evolution of common metabolisms. Alternatively, the similarity of these enzymes, which would have been involved in ancestral common metabolism, may be remnants of enzymes lost in many other present lineages.

Similarly, the biosynthesis of diosgenin, a specialized steroidal saponin derived from triterpenes, is known to undergo convergent evolution in different lineages—dicotyledonous plants, including Leguminosae and Solanaceae, and monocotyledonous plants including Meliaceae and Dioscoreaceae (**Table 2**). It has been reported that in both monocots and dicots, cholesterol dihydroxylation reactions are commonly catalyzed by enzymes of the CYP90 family, but the subsequent monohydroxylation and cyclization reactions are catalyzed by distinct subclasses of CYP monooxygenases (Christ et al. 2019). The dihydroxylation of cholesterol is a reaction similar to brassinosteroid biosynthesis, suggesting that the dihydroxylases evolved independently from a common ancestral CYP gene for brassinosteroids.

These pioneering works described earlier indicate that convergent metabolic evolution has likely occurred repeatedly in various classes of enzyme genes. Metabolic evolution is basically achieved by duplicating and modifying preexisting enzymes as the *de novo* design of enzyme genes to catalyze specific reactions for specific substrates is extremely difficult; it is much easier to change the substrate specificity, regiospecificity or sugar-donor selectivity of preexisting enzymes. Given the cellular ability to copy genes with catalytic preferences, it is reasonable that altering the function of copied genes increases the variation and therefore the chance for convergence in metabolic evolution. However, lineage-specific recruited enzymes partly reflect differences in genetic availability, presumably owing to the specialized genomic preset associated with speciation.

## Multivalent Metabolites

Specialized metabolites generally refer to phylogenetically specific small-molecule compounds. A broad spectrum of structural diversity in specialized metabolites has long been regarded as the basis for the unique biological activities exhibited by each specialized metabolite with unique chemical structures. However, emerging evidence shows that there are cases where metabolites that are conserved across kingdoms display distinct biological activities in species from different kingdoms. We propose to define such metabolites as ‘multivalent metabolites’. It is noteworthy that a group of small molecules in animals, long known as neurotransmitters, are often widely conserved in plants and microorganisms and have characteristic bioactivities outside the animal kingdom. Examples of such molecules include amino acids and biogenic monoamines. In plants, amino acids and biogenic monoamines are often part of the specific response to disease, mechanical wounding and drought stress. For example, the nonprotein amino acid gamma-aminobutyric acid, which is an inhibitory neurotransmitter in the central nervous system of mammals, has been suggested to be a signaling molecule involved in drought stress and disease response in plants (Bown and Shelp 2016). Moreover, the amino acid glutamate, which also functions as a neurotransmitter in animals, acts as a long-range signaling agent across organs in plants (Toyota et al. 2018). Acetylcholine (ACh), which was identified as the first example of a neurotransmitter, is also ubiquitous in plants. The biological function of ACh in plants has been largely attributed to responses against environmental stresses including (but not limited to) salinity (Qin et al. 2021). *A. thaliana* is capable of biosynthesizing ACh *in planta*, and the exogenous application of ACh has been shown to promote root hair development (Murata et al. 2015). The higher accumulation of ACh in *Solanum melongena* (eggplant) and *Phyllostachys* spp. (bamboo) than mammalian neuronal tissues implies that ACh might have untapped biological functions in these plant species (Horiuchi et al. 2003). In addition, biogenic monoamines constitute a group of specialized metabolites sporadically yet broadly found in all three kingdoms of life and exhibit biological activities that are unique to each species. Serotonin, known as the ‘happy hormone’ in animals, was shown to be incorporated into the cell wall and participates in the containment of the pathogen *Bipolaris oryzae* in *Oryza sativa* (rice) (Ishihara et al. 2008, Fujiwara et al. 2010). Melatonin, which has long been known as a neuronal hormone that regulates the sleep–wake cycle in mammals, is also found in plants, and its biological function has often been associated with disease resistance (Back 2021). Histamine, which functions both as a neurotransmitter and as a signaling molecule in the local immune response in animals, has been shown to accumulate extensively in the stinging hairs of *Urtica* spp. (nettle) and other selected plant species and is thought to protect the plants from herbivores by inducing itching and inflammation in animals (Iwamoto et al. 2014, Ensikat et al. 2021). Notably, the discovery of histamine and ACh was not achieved from animal tissues but from rye infected with ergot fungi (Barger and Dale 1910). Short-chain fatty acids (SCFAs) are another class of small molecules that can be categorized as ‘multivalent metabolites’. Recent studies show that SCFAs might serve more than just as intermediates in carbon metabolism and additionally as environmental signaling molecules that are monitored by various organisms. For example, butyrate produced by gut microbes triggers macrophage-mediated immune responses under anaerobic conditions in the mammalian intestine (Chang et al. 2014). In plants, endogenous acetic acid promotes tolerance against drought stress, and isovaleric acid from *Bacillus* spp. has been shown to trigger growth inhibition (Kim et al. 2017, Murata et al. 2022). SCFAs are often converted into esters of coenzyme A (CoA) and then serve as substrates for the acylation of lysine residues of histone in eukaryotes, showing that SCFAs are indirectly involved in epigenetic regulation (Nitsch et al. 2021). The CoA ester of 2-hydroxyisobutyrate, among other SCFAs, has been associated with the dark-induced metabolic shift in sugar metabolism in plants (Zheng et al. 2021). As the perception of free-acid forms of SCFAs by plants is largely unknown, it is unclear whether SCFAs exhibit their regulatory activities either directly as free acids or indirectly as substrates, for example, for histone modification regulators. Amino acids, biogenic amines and SCFAs are universally present across kingdoms and thus have not been necessarily classified as specialized metabolites from an authentic metabolite categorization. However, the emerging biological roles of these metabolites in plants suggest that, in addition to small molecules with unique structures and biological functions, small molecules with highly conserved structures across kingdoms exhibiting lineage-specific physiological functions in plants could also be included in the list of plant specialized metabolites. Therefore, the definition of plant specialized metabolites might require minor modification.

While the chemical structures of multivalent metabolites are highly conserved in animals and plants, the biosynthetic mechanisms of those metabolites are unique to plants in some instances. For example, serotonin in animals is formed through the hydroxylation of tryptophan at the C5 position, followed by decarboxylation of the carboxyl group. In contrast, plants biosynthesize serotonin through the decarboxylation of tryptophan, followed by hydroxylation at the C5 position (Fujiwara et al. 2010). ACh, in contrast, is generated by transferring an acetyl group from acetyl-CoA to choline by choline-*O*-acetyltransferase (ChAT) in animals. However, no genes with substantial sequence similarity to the previously identified animal ChAT have been found in plants, leaving the biosynthetic origin of plant ACh ambiguous. Instead, an esterase that hydrolyzes ACh was isolated from maize and has been shown to belong to a plant-specific GDSL lipase family (Sagane et al. 2005) that is likely to be phylogenetically specific, as the corresponding enzyme activity was not detected from its orthologous gene in *A. thaliana* (Muralidharan et al. 2013). Similarly, the sporadic occurrence of serotonin and melatonin in plants can be explained primarily from the findings that only selected plant species have genes encoding tryptophan decarboxylase (TDC), a branch-point enzyme that converts tryptophan into tryptamine, which is a central precursor to serotonin, melatonin and many other specialized metabolites with an indole moiety, including a spectrum of monoterpenoid indole alkaloids (Negri et al. 2021). Notably, genes encoding plant TDC do not show apparent sequence similarity to those of animals and have so far been functionally identified only in selected species including *Catharanthus roseus* (vinca) and *Ophiorrhiza pumila* (De Luca et al. 1989, Yamazaki et al. 2003). Moreover, the sequence similarity among those functionally characterized plant TDC genes is approximately 70% at the amino acid level (Negri et al. 2021). Collectively, these reports indicate that, as in the evolutionary scenario of ‘authentic’ plant specialized metabolites, both convergent evolution and lineage-specific parallel evolution likely play key roles in the sporadic occurrence of multivalent metabolites in various plant species.

## Co-presence and Metabolon

The biosynthesis of specialized metabolites generally involves multiple enzymes for the completion of the pathway. Thus, in addition to the catalytic specificities of enzymes involved, (i) the synchronous spatial and temporal gene expression and (ii) the co-localization of enzymes are essential for achieving the proper operation of plant specialized metabolism (Chae et al. 2014). The transcriptional co-expression of enzyme genes in plant specialized metabolism is partly supported by gene clustering that has occurred as a consequence of gene multiplication. However, the co-expression mechanisms of enzyme genes that are located in a discrete locus of the genome remain largely elusive. In turn, the orthotopic nature of enzyme gene expression in plant specialized metabolism allowed Arabidopsis thaliana trans factor and cis-element prediction database II (ATTED-II) and other co-expression analyses to be implemented for identifying a number of enzyme genes (Itkin 2013, Shang et al. 2014, Lau and Sattely 2015, Ono et al. 2020, Obayashi et al. 2022).

The continuous reactivity of oxidation and glycosylation likely contributed to the enhanced coupling of CYP/DOX and UGT. As reported in Solanaceous tobacco, a defect in a UGT leads to deglycosylation and the accumulation of toxic aglycones, resulting in impaired growth (Heiling et al. 2021). Therefore, adaptation to endogenous metabolic disorders while coping with fluctuating environmental cues is a central issue in the development of new metabolism; the molecular evolution of UGT as partner enzymes with CYPs and DOXs is likely to be key to minimizing the potential risks of autotoxicity from newly synthesized metabolites (Li et al. 2021). Indeed, along with the evolution of plant genomes, the copy number of CYP/DOX and UGT superfamily enzyme genes would have increased coordinately in seed plants (Kawai et al. 2014), suggesting that avoiding autotoxicity is a key constraint to the specialized metabolic evolution.

In contrast, metabolons that are putative enzyme complexes have garnered increasing attention as a possible mechanism for optimizing sequential metabolic reactions (Pandey et al. 2017). A metabolon is essentially an enzyme–protein complex that is integrated into organelle membranes and is thought to channel sequential enzyme reactions, thereby facilitating and optimizing the overall metabolic reaction cascades while avoiding the reactivity and toxicity of intermediate metabolites by metabolic channeling. The physical interaction is the most apparent case of the co-presence of proteins in the vicinity, enabling catalytic cooperation and substrate channeling through micro-locally enriched metabolic fluxes in subcellular compartments (Zhang and Fernie 2021). The formation of metabolons is known in conserved core metabolic pathways of prokaryotic and eukaryotic cells, including glycolytic bodies in which the enzymes of the glycolytic system assemble under low oxygen conditions, the tricarboxylic acid cycle, Calvin–Benson cycle and nucleotide synthesis by the purinosome (Schmitt and An 2017, Zhang et al. 2017, Pareek et al. 2021).

Metabolons play a role not only in core metabolism but also in specialized metabolism (Nakayama et al. 2019, Zhang and Fernie 2021). Metabolons that catalyze the biosynthesis of cyanogenic glycosides and lignan glycosides (Ono et al. 2020) are additional examples of metabolons composed of CYP and UGT with CYP reductase (CPR) (Laursen et al. 2016). During the metabolic pathway, specialized metabolites are often oxidized and further decorated with sugar moieties to form *O-*glycosides, many of which are thought to be catalyzed by CYP and UGT. Generally, the glycosylation of aglycones contributes to enhanced water solubility and reduced substrate reactivity. The rich repertoire of CYP and UGT genes in plant genomes serves as the physical and biochemical core of metabolons and should allow for evolving metabolons with new catalytic activity.

Given that CYP and UGT are highly multiplied enzymes frequently involved in specialized metabolism (Kawai et al. 2014), we speculate that they would have been partly optimized as ready-to-interact as metabolon components during plant metabolic evolution. Notably, the formation of metabolon between CYP and UGT is also observed in mammalian phase II xenobiotic metabolism; the regio-selectivity of morphine glucuronidation by UGT2B7 was found to be altered by specific interaction with CYP3A4 (Ishii et al. 2007). The physical interaction between CYPs and UGTs commonly observed in plant and animal kingdoms suggests the biological importance of the interaction between CYP and UGT in continuous reactions of oxidation followed by glycosylation. Therefore, the interaction of metabolic enzymes is a prerequisite—not only in avoiding autotoxicity by providing physical channeling of substrate-binding pockets and rapid detoxification by attaching sugar moieties but also in biochemical cooperation in their catalysis (Tatsis et al. 2017, Heiling et al. 2021, Li et al. 2021).

More recently, more studies have reported that pathway enzymes are not the only components of metabolons; there are also proteins that provide scaffolds for enzyme complexes and maintain and control the structural integrity of metabolons (Gou et al. 2018, Waki et al. 2020). Understanding the importance of metabolons should lead to a better overview of the molecular mechanism that drives the biochemical diversity in specialized metabolism during evolution.

It has also been recently recognized that soluble enzymes and small molecules can be confined to a micro-environment by liquid–liquid phase separation without the presence of a membrane system, thereby improving the efficiency of enzyme reactions (Dahmani et al. 2023). Notably, when expressed in yeast and hetero-multimerized through an optogenetic approach, violacein biosynthetic cluster genes VioC and VioE preferentially catalyzed the formation of an antibacterial and antifungal alkaloid, deoxyviolacein, from protodeoxyviolaceinate, which is otherwise easily oxidized nonenzymatically to prodeoxyviolacein (Zhao et al. 2019). The results clearly indicate the feasibility of liquid–liquid phase separation as an alternative mechanism to metabolons for enhancing the metabolic reactions. Although the optimization of the metabolic pathway likely depends on metabolons for membrane-associated enzymes and their interactors, liquid–liquid phase separation might play an indispensable role in the efficacy of the metabolic pathway exclusively involving soluble enzymes. In both cases, the reconstitution of the reactions and the establishment of a quantitative assessment system for efficacies of enzyme reactions are essential for the proof-of-concept studies.

## Specialized Metabolism in Domestication

Why do plants produce structurally diverse specialized metabolites? A general explanation is that specialized metabolites increase the ecological fitness of plants. For example, flower pigments, scents and toxins help to attract or repel certain species (Arimura et al. 2009). The biological activities of specialized metabolites have been studied within an ecological context in nature and were considered to help maximize the chance of survival following various biotic interactions with pollinators, seed dispersers, pathogens and commensal fungi, as well as experiencing drought, UV or other abiotic stresses. However, the biological functions of specialized metabolites in plants, especially under the laboratory setup and in experimental fields, are often ambiguous. This is because the biological functions of metabolites have been originally optimized for plants in natural habits. Therefore, metabolites can easily become less valuable under artificially controlled environments wherein the fluctuation in various biotic and abiotic environmental parameters is less than that in natural ecosystems. There are pioneering examples of metabolite functions from an ecological context associated with pollinator preference shift via the biosynthesis of various specialized metabolites (e.g. anthranilates, flavonoids, carotenoids and alkaloids) affecting recognizable phenotypes in floral petals, anthers and nectars, which likely affect reproductive isolation and eventually speciation (Lüthi et al. 2022, Roy et al. 2022, Liang et al. 2023, Mori et al. 2023). Nevertheless, the vast majority of the ‘extended’ phenotypes would not have been verified unless the plants were placed in the correct ecological context to exert specialized functions.

In contrast to wild species living in natural habitats, cultivated crops are organisms that have been developed to support human life. Together with productivity, which is the most important agronomic trait, specialized metabolites that contribute to commercial traits in quality—color, aroma, taste or storage durability extending shelf life—have also been intensively selected during domestication and modern breeding. Historically, flower color variants of horticultural plants, e.g. tulips and morning glories, have been bred and collected. Collections of germplasms of snapdragon (*Antirrhinum*) and morning glory (*Ipomoea*) contributed to the understanding of the flavonoid biosynthetic pathway and acted as a genetic resource for identifying various flavonoid biosynthetic genes (Rausher et al. 1999, Bradley et al. 2017).

Similarly, specialized metabolites causing bitterness, astringency and toxicity, which were often found in the edible parts of ancestors of modern crops, have been selectively removed by sensory screening. For example, genomic comparisons of the biosynthetic gene clusters in cucumber cucurbitacins and almond cyanogenic glycosides revealed that the corresponding functional biosynthetic genes present in wild species are absent from modern crops (Shang et al. 2014, Zhou et al. 2016, Sánchez-Pérez et al. 2019). Similarly, as the terminal sugar modification in group A soyasaponins is associated with strong bitterness and astringency, the removal of the responsible UGT genes has been a breeding target for improving the aftertaste of soybean products (Sayama et al. 2012).

Moreover, the levels of capsaicinoids (pungent components in chili pepper) and caffeine (alkaloid stimulant in tea) have been manipulated (Ogino et al. 2019, Tanaka et al. 2019). In the case of red wine, astringency is a positive trait suitable for long-term aging. The Tannat cultivar of red wine grapes is famous for an extremely high polyphenol content; accordingly, the cultivar-specific polyphenol biosynthetic enzyme gene is enriched in the genome compared with the Pinot Noir cultivar (Da Silva et al. 2013). This is thought to be a result of artificial selection of an anthocyanin- and tannin-rich cultivar.

In the case of tomato, enzyme genes that are involved in the formation of a bitter substance, tomatine, and a smoky volatile, guaiacol, have been either deleted or modified to accumulate as static glycosides by the introduction of new hydroxylation and sugar modifications (Tikunov et al. 2013, Cárdenas et al. 2019, Kazachkova et al. 2021, Akiyama et al. 2021a, 2022). These reports show that bitterness and toxicity have been reduced in various crops during domestication and that specialized metabolism can be readily altered with sufficient selective pressure. Even prior to the age of molecular biology and functional genomics, without modern transgenic technologies, humans modified the specialized metabolites of domesticated crops by selecting favorable sensory traits for dietary foods and beverages. These specialized metabolites would have contributed to ecological fitness in the native environment, but their functions are evaluated in an agricultural context and appear to be partly substituted or enforced by pesticides and fertilizers under artificial cultivation.

## Evolvability of Specialized Metabolism

The molecular mechanism of how enzymes have acquired new functions is poorly understood. The escape of adaptive conflict theory predicts that the emergence of the novel catalytic activity that accepts originally unaccepted metabolites and produces minor metabolites precedes gene duplication (Des Marais and Rausher 2008); thus, the newly acquired catalytic activity will be enhanced when the minor metabolites enhance an individual’s fitness. Gene duplication appears not only to solve the physicochemical dilemma of functional constraints between the original and the new activity of a single progenitor enzyme and allow the new activity to be rewired within a reasonable spatiotemporal framework but also to secure a molecular basis for the swift evolution of metabolic pathways (Lanier et al. 2023). Indeed, it is known that the catalytic activity of an enzyme often increases when the promiscuous catalytic activity toward multiple substrates becomes specific to a single substrate owing to the negative trade-off between catalytic promiscuity (generalism) and specificity (specialism) (Des Marais and Rausher 2008, Khersonsky and Tawfik 2010). Thus, the latent and promiscuous activities of enzymes are crucial seeds for metabolic evolution. It is important to note that when the duplicated genes are rewired to be expressed in different spatiotemporal locations, biochemical adaptation of such genes is placed in new metabolic contexts. This would liberate the enzymes from biochemical constraints for maintaining originally assigned catalytic activities and allow them to readily become promiscuous until acquiring new biochemical functions. However, given the limited information on known catalytic activities of the vast majority of enzymes, the catalytic modulation by tissue-specific physical interactions of catalytic enzymes, noncatalytic scaffold proteins and redox partners and allostery by protein–metabolite interactions (Tatsis et al. 2017, Baker and Rutter 2023, Zhao et al. 2023), we understand only a very small part of the metabolic evolvability that enables the development of the huge chemical diversity of specialized metabolites in nature.

Many specialized metabolic genes are frequently duplicated in tandem at specific genomic locations (Chae et al. 2014) although some are known to be duplicated by retroposition from transcripts into the genome (Matsuno et al. 2009). Importantly, there have also been reports of metabolic evolution in eukaryotes via horizontal gene transfer (HGT) (Kirsch et al. 2022). Phenylalanine ammonia lyase, the enzyme catalyzing the first committed step in the phenylpropanoid pathway leading to lignin, lignan and flavonoids, was acquired ancestrally via HGT during symbiosis with soil bacteria (Emiliani et al. 2009). Thus, it is of particular interest whether HGT has played indispensable roles in the evolution of specialized metabolism in plants.

Older genes, such as those involved in central metabolism, are intertwined with many intermolecular optimizations, and it has been reported that spare genes are rarely retained before they acquire new gene functions due to high molecular entanglements (Kuzmin et al. 2020). The functional differentiation of duplicated genes has been studied, but the evolution of new functions has been reported to be highly related to the low potency of the underlying gene. This is likely because many enzymes that mediate specialized metabolism are lineage-specific (i.e. recently) multiple superfamily genes that successfully developed different catalytic activities. Because groups of genes that have formed relatively recently, such as those for specialized metabolism, are usually distant from genes involved in central metabolism, it is unlikely that they have experienced a high degree of intermolecular optimization with other genes compared to central metabolic genes. Therefore, they may be more likely to undergo functional innovation. The unique evolutionary context located at the periphery of metabolism allows the emergence of highly specialized metabolic functions via the low entanglement of catalytic units (**Table 1**).

**Fig. 3:**
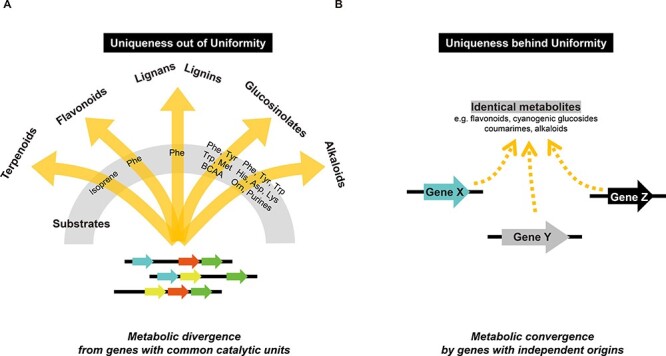
Two conceptual modes that support the evolvability of plant specialized metabolism. (A) The ‘uniqueness out of uniformity’ concept. Biosynthesis of plant specialized metabolites generally starts from highly central and highly conserved (i.e. across the plant kingdom) core metabolites as precursors. Therefore, the vast structural diversity of plant specialized metabolites depends largely on an array of catalytic properties exhibited by specific enzymes. However, such enzymes often feature common catalytic units that drive metabolic divergence. (B) The ‘uniqueness behind uniformity’ concept. On the other hand, there are examples of common plant specialized metabolites that are biosynthesized by a specific set of enzyme-coding genes that do not share an apparent common evolutionary origin. The trajectories to assemble specialized metabolisms can be likened to “bricolage”.

Steviol glycosides that are widely used as natural sweeteners are derived from the metabolism of diterpenes, which share their biosynthetic origins with the phytohormone gibberellin. Likewise, both triterpene saponins/steroidal glycoalkaloids and brassinosteroids are derived from shared sterol precursors. Moreover, auxins and glucosinolates are derived from tryptophan, whereas strigolactones originate from carotenoids. The biosynthesis and metabolism of all of these phytohormones are also mediated by many oxygenation and glycosylation reactions, practically sharing the involvement of CYP, DOX and UGT genes with specialized metabolism, as discussed in this review. We speculate that these genes were unlikely recruited to various metabolic pathways on the basis of the high gene multiplicity in plant genomes but rather that they became multiple genes as the common catalytic units for the evolutionary latency to assemble metabolism at multiple levels of co-presence; common transcription factors (Shoji 2019), protein–protein interactions (Nakayama et al. 2019, Zhang and Fernie 2021) and subcellular compartmentation by phase separation (Dahmani et al. 2023) should have played indispensable roles in the molecular evolution of catalytic units with unique biochemical properties. In other words, these catalytic units are specialized in that they are prone to reorganize new metabolism. The uniformity in cooperative and low entangled catalytic units would ultimately allow for thrifty natural selection, avoiding the much costly *de novo* synthesis of such units (**Fig. 3**). Therefore, it is feasible that particular gene families have been expanded through feedforward interactions, in which the repurposed units are functionally optimized (**Table 1**). Metabolism is actually a continuous process, with no clear boundaries between categories, but the features described here provide a new perspective for understanding metabolic evolution in plants.

Plants are constantly updating their specialized metabolism to increase ecological fitness for their survival in nature. Therefore, specialized metabolic evolution is an arms race for adaptive chemical traits by diversifying common enzyme genes, which is analogous to the race against pathogen effectors via the diversification of multiple nucleotide-binding domains and leucine-rich repeat-containing gene (NLR)-mediated plant immunity (Jones et al. 2016). They are comparable in terms of the race for diversification of interacting molecules that function at the boundary between organisms. Even the biological relevance of the metabolites that are currently crucial for a plant will be biochemically updated as the environmental context changes. However, the manner of updating the bioactivities of the metabolites might not significantly change compared with the ongoing evolution of specialized metabolisms. The accumulation of this structure–activity relationship will help predict enzyme activity and the convergent evolution of enzymes in specialized metabolism (Yang et al. 2018, Fukushima and Pollock 2022).

## Perspectives from the Underground

In this review, we described possible mechanisms of how the convergent evolution, co-presence and evolvability of specialized metabolisms have been achieved. Driving force to convergently develop the identical metabolites currently remain unknown. We speculate that the biological activities of convergent specialized metabolites in analogous tissues and organs in different plants are likely to be associated with the adaptations for disease resistance, microbial symbiosis, pollinator attraction and herbivore avoidance that are commonly indispensable among various plant species. However, this is not the case when the sites of accumulation of the identical metabolites are distinct in different plant species. Aurone found in nonflowering liverworts is speculated to contribute to UV tolerance on land. However, in flowering plants, aurone pigments may also contribute to pollinator attraction via coloration in floral organs (Davies et al. 2020). Thus, specialized metabolites may have different physiological functions in different evolutionary contexts. For example, the *in planta* role of sesamin has long been enigmatic. However, the recent discovery of soil-borne microorganisms that have acquired sesamin-degrading enzymes from sesame fields suggests that sesamin is consumed by selected microorganisms (Kumano et al. 2016). Similarly, caffeine-degrading microbes have been identified (Summers et al. 2015). It would be particularly interesting to clarify whether these microbes that evolved to assimilate specialized metabolites are enriched in cultivated fields of crops producing the responsible specialized metabolites for the sake of hidden biological interactions.

Specialized metabolites secreted from roots include triterpenoid acids and coumarins, which are thought to participate in interactions with soil microorganisms and insects (Nakayasu et al. 2022, Stringlis et al. 2019; Zhong et al. 2022). Findings have emerged that plants promote specific microbiota formation, via specialized metabolites, to obtain water, minerals, and organic compounds. Future studies will clarify whether specialized metabolites with unknown functions play important roles in symbiosis and co-evolution with other underground organisms as in the case of thalianol and other specialized metabolites that are secreted from the roots of *A. thaliana* (Huang et al. 2019). Furthermore, several intestinal microorganisms that metabolize ingested plant specialized lignans into enterolignans have been reported in the human gut microbiome (Bess et al. 2020), providing a new ecological perspective on external biological interactions via specialized metabolites with the microbiome beyond the internal physiology of plants producing specialized metabolites. Thus, specialized metabolism is expected to expand into the large field of the metabolite-mediated interplay between multiple organisms, from ecology and agriculture to human health.

## Supplementary Material

pcad057_Supp

## Data Availability

The data for metabolite (**Fig. 1A**) and enzymes (**Fig. 1B**) are available in KNApSAcK database at http://www.knapsackfamily.com/KNApSAcK_Family/ and UniProt Knowledgebase at https://www.uniprot.org/uniprotkb?query=*, respectively.

## References

[R1] Afendi F.M., Okada T., Yamazaki M., Hirai-Morita A., Nakamura Y., Nakamura K., et al. (2012) KNApSAcK family databases: integrated metabolite-plant species databases for multifaceted plant research. *Plant Cell Physiol.* 53: 1–12. 22123792 10.1093/pcp/pcr165

[R2] Akiyama R., Nakayasu M., Umemoto N., Kato J., Kobayashi M., Lee H.J., et al. (2021a) Tomato E8 encodes a C-27 hydroxylase in metabolic detoxification of α-tomatine during fruit ripening. *Plant Cell Physiol.* 62: 775–783. 34100555 10.1093/pcp/pcab080

[R3] Akiyama R., Watanabe B., Kato J., Nakayasu M., Lee H.J., Umemoto N., et al. (2022) Tandem gene duplication of dioxygenases drives the structural diversity of steroidal glycoalkaloids in the tomato clade. *Plant Cell Physiol.* 63: 981–990. 35560060 10.1093/pcp/pcac064

[R4] Akiyama R., Watanabe B., Nakayasu M., Lee H.J., Kato J., Umemoto N., et al. (2021b) The biosynthetic pathway of potato solanidanes diverged from that of spirosolanes due to evolution of a dioxygenase. *Nat. Commun.* 12: 6–15. 33637735 10.1038/s41467-021-21546-0 PMC7910490

[R5] Arimura G.I., Matsui K. and Takabayashi J. (2009) Chemical and molecular ecology of herbivore-induced plant volatiles: proximate factors and their ultimate functions. *Plant Cell Physiol.* 50: 911–923. 19246460 10.1093/pcp/pcp030

[R6] Back K. (2021) Melatonin metabolism, signaling and possible roles in plants. *Plant J.* 105: 376–391. 32645752 10.1111/tpj.14915

[R7] Bai Y., Fernández-Calvo P., Ritter A., Huang A.C., Morales-Herrera S., Bicalho K.U., et al. (2021) Modulation of *Arabidopsis* root growth by specialized triterpenes. *New Phytol.* 230: 228–243. 33616937 10.1111/nph.17144

[R8] Baker S.A. and Rutter J. (2023) Metabolites as signalling molecules. *Nat. Rev. Mol. Cell Biol.* 24: 355–374. 36635456 10.1038/s41580-022-00572-w

[R9] Barger G. and Dale H.H. (1910) Chemical structure and sympathomimetic action of amines. *J. Physiol.* 41: 19–59. 16993040 10.1113/jphysiol.1910.sp001392 PMC1513032

[R10] Beran F., Köllner T.G., Gershenzon J. and Tholl D. (2019) Chemical convergence between plants and insects: biosynthetic origins and functions of common secondary metabolites. *New Phytol.* 223: 52–67. 30707438 10.1111/nph.15718

[R11] Berland H., Albert N.W., Stavland A., Jordheim M., McGhie T.K., Zhou Y., et al. (2019) Auronidins are a previously unreported class of flavonoid pigments that challenges when anthocyanin biosynthesis evolved in plants. *Proc. Natl. Acad. Sci. USA* 116: 20232–20239. 31527265 10.1073/pnas.1912741116 PMC6778211

[R12] Berman P., de Haro L.A., Jozwiak A., Panda S., Pinkas Z., Dong Y., et al. (2023) Parallel evolution of cannabinoid biosynthesis. *Nat. Plants* 9: 817–831. 37127748 10.1038/s41477-023-01402-3

[R13] Bess E.N., Bisanz J.E., Yarza F., Bustion A., Rich B.E., Li X., et al. (2020) Genetic basis for the cooperative bioactivation of plant lignans by *Eggerthella lenta* and other human gut bacteria. *Nat. Microbiol.* 5: 56–66. 31686027 10.1038/s41564-019-0596-1 PMC6941677

[R14] Bown A.W. and Shelp B.J. (2016) Plant GABA: not just a metabolite. *Trends Plant Sci.* 21: 811–813. 27542324 10.1016/j.tplants.2016.08.001

[R15] Boyce G.R., Gluck-Thaler E., Slot J.C., Stajich J.E., Davis W.J., James T.Y., et al. (2019) Psychoactive plant- and mushroom-associated alkaloids from two behavior modifying cicada pathogens. *Fungal Ecol.* 41: 147–164. 31768192 10.1016/j.funeco.2019.06.002 PMC6876628

[R16] Bradley D., Xu P., Mohorianu I.I., Whibley A., Field D., Tavares H., et al. (2017) Evolution of flower color pattern through selection on regulatory small RNAs. *Science* 358: 925–928. 29146812 10.1126/science.aao3526

[R17] Brahmachari G., Mandal L.C., Roy R., Mondal S. and Brahmachari A.K. (2011) Stevioside and related compounds - molecules of pharmaceutical promise: a critical overview. *Arch. Pharm. Chem. Life Sci.* 344: 5–19. 10.1002/ardp.201000181 21213347

[R18] Cárdenas P.D., Sonawane P.D., Heinig U., Jozwiak A., Panda S., Abebie B., et al. (2019) Pathways to defense metabolites and evading fruit bitterness in genus Solanum evolved through 2-oxoglutarate-dependent dioxygenases. *Nat. Commun.* 10: 1–13. 31727889 10.1038/s41467-019-13211-4 PMC6856131

[R19] Chae L., Kim T., Nilo-Poyanco R. and Rhee S.Y. (2014) Genomic signatures of specialized metabolism in plants. *Science* 344: 510–513. 24786077 10.1126/science.1252076

[R20] Chang P.V., Hao L., Offermanns S. and Medzhitov R. (2014) The microbial metabolite butyrate regulates intestinal macrophage function via histone deacetylase inhibition. *Proc. Natl. Acad. Sci. USA* 111: 2247–2252. 24390544 10.1073/pnas.1322269111 PMC3926023

[R21] Cho M.H., Moinuddin S.G., Helms G.L., Hishiyama S., Eichinger D., Davin L.B., et al. (2003) (+)-Larreatricin hydroxylase, an enantio-specific polyphenol oxidase from the creosote bush (*Larrea tridentata*). *Proc. Natl. Acad. Sci. USA* 100: 10641–10646. 12960376 10.1073/pnas.1934562100 PMC196857

[R22] Christ B., Xu C., Xu M., Li F.S., Wada N., Mitchell A.J., et al. (2019) Repeated evolution of cytochrome P450-mediated spiroketal steroid biosynthesis in plants. *Nat. Commun.* 10: 1–11. 31324795 10.1038/s41467-019-11286-7 PMC6642093

[R23] Dahmani I., Qin K., Zhang Y. and Fernie A.R. (2023) The formation and function of plant metabolons. *Plant J.* 114: 1080–1092. 36906885 10.1111/tpj.16179

[R24] Da Silva C., Zamperin G., Ferrarini A., Minio A., Dal Molin A., Venturini L., et al. (2013) The high polyphenol content of grapevine cultivar tannat berries is conferred primarily by genes that are not shared with the reference genome. *Plant Cell* 25: 4777–4788. 24319081 10.1105/tpc.113.118810 PMC3903987

[R25] Davies K.M., Jibran R., Zhou Y., Albert N.W., Brummell D.A., Jordan B.R., et al. (2020) The evolution of flavonoid biosynthesis: a bryophyte perspective. *Front. Plant Sci.* 11: 1–21. 32117358 10.3389/fpls.2020.00007 PMC7010833

[R26] De Luca V., Marineau C. and Brisson N. (1989) Molecular cloning and analysis of cDNA encoding a plant tryptophan decarboxylase: comparison with animal dopa decarboxylases. *Proc. Natl. Acad. Sci. USA* 86: 2582–2586. 2704736 10.1073/pnas.86.8.2582 PMC286961

[R27] Des Marais D.L. and Rausher M.D. (2008) Escape from adaptive conflict after duplication in an anthocyanin pathway gene. *Nature* 454: 762–765. 18594508 10.1038/nature07092

[R28] de Vries S., Fürst-Jansen J.M.R., Irisarri I., Dhabalia Ashok A., Ischebeck T., Feussner K., et al. (2021) The evolution of the phenylpropanoid pathway entailed pronounced radiations and divergences of enzyme families. *Plant J.* 107: 975–1002. 34165823 10.1111/tpj.15387

[R29] Emiliani G., Fondi M., Fani R. and Gribaldo S. (2009) A horizontal gene transfer at the origin of phenylpropanoid metabolism: a key adaptation of plants to land. *Biol. Direct* 4: 1–12. 19220881 10.1186/1745-6150-4-7 PMC2657906

[R30] Ensikat H., Wessely H., Engeser M. and Weigend M. (2021) Distribution, ecology, chemistry and toxicology of plant stinging hairs. *Toxins (Basel)* 13: 141. 10.3390/toxins13020141 PMC7918447 33668609

[R31] Fujiwara T., Maisonneuve S., Isshiki M., Mizutani M., Chen L., Ling Wong H., et al. (2010) Sekiguchi lesion gene encodes a cytochrome P450 monooxygenase that catalyzes conversion of tryptamine to serotonin in rice. *J. Biol. Chem.* 285: 11308–11313. 20150424 10.1074/jbc.M109.091371 PMC2857009

[R32] Fukushima K. and Pollock D.D. (2022) Detecting macroevolutionary genotype-phenotype associations using error-corrected rates of protein convergence. *Nat. Ecol. Evol.* 7: 155–170. 10.1038/s41559-022-01932-7 PMC9834058 36604553

[R33] Furudate H., Manabe M., Oshikiri H., Matsushita A., Watanabe B., Waki T., et al. (2023) A polyphenol oxidase catalyzes aurone synthesis in *Marchantia polymorpha*. *Plant Cell Physiol.* pcad024. 10.1093/pcp/pcad024 36947436

[R34] Gou M., Ran X., Martin D.W. and Liu C.J. (2018) The scaffold proteins of lignin biosynthetic cytochrome P450 enzymes. *Nat. Plants* 4: 299–310. 29725099 10.1038/s41477-018-0142-9

[R35] Hansen C.C., Nelson D.R., Møller B.L. and Werck-Reichhart D. (2021) Plant cytochrome P450 plasticity and evolution. *Mol. Plant* 14: 1244–1265. 34216829 10.1016/j.molp.2021.06.028

[R36] Hansen C.C., Sørensen M., Bellucci M., Brandt W., Olsen C.E., Goodger J.Q.D., et al. (2022) Recruitment of distinct UDP‐glycosyltransferase families demonstrates dynamic evolution of chemical defense within Eucalyptus L’Hér . *New Phytol.* 237: 999–1013. 36305250 10.1111/nph.18581 PMC10107851

[R37] Heiling S., Llorca L.C., Li J., Gase K., Schmidt A., Schafer M., et al. (2021) Specific decorations of 17-hydroxygeranyllinalool diterpene glycosides solve the autotoxicity problem of chemical defense in *Nicotiana attenuata*. *Plant Cell* 33: 1748–1770. 33561278 10.1093/plcell/koab048 PMC8254506

[R38] Hirai N., Yoshida R., Todoroki Y. and Ohigashi H. (2000) Biosynthesis of abscisic acid by the non-mevalonate pathway in plants, and by the mevalonate pathway in fungi. *Biosci. Biotechnol. Biochem.* 64: 1448–1458. 10945263 10.1271/bbb.64.1448

[R39] Hong B., Grzech D., Caputi L., Sonawane P., López C.E.R., Kamileen M.O., et al. (2022) Biosynthesis of strychnine. *Nature* 607: 617–622. 35794473 10.1038/s41586-022-04950-4 PMC9300463

[R40] Horiuchi Y., Kimura R., Kato N., Fujii T., Seki M., Endo T., et al. (2003) Evolutional study on acetylcholine expression. *Life Sci.* 72: 1745–1756. 12559395 10.1016/s0024-3205(02)02478-5

[R41] Huang A.C., Jiang T., Liu Y.X., Bai Y.C., Reed J., Qu B., et al. (2019) A specialized metabolic network selectively modulates *Arabidopsis* root microbiota. *Science* 364: aau6389. 10.1126/science.aau6389 31073042

[R42] Huang R., O’Donnell A.J., Barboline J.J. and Barkman T.J. (2016) Convergent evolution of caffeine in plants by co-option of exapted ancestral enzymes. *Proc. Natl. Acad. Sci. USA* 113: 10613–10618. 27638206 10.1073/pnas.1602575113 PMC5035902

[R43] Ishihara A., Hashimoto Y., Tanaka C., Dubouzet J.G., Nakao T., Matsuda F., et al. (2008) The tryptophan pathway is involved in the defense responses of rice against pathogenic infection via serotonin production. *Plant J.* 54: 481–495. 18266919 10.1111/j.1365-313X.2008.03441.x

[R44] Ishii Y., Iwanaga M., Nishimura Y., Takeda S., Ikushiro S.I., Nagata K., et al. (2007) Protein-protein interactions between rat hepatic cytochromes P450 (P450s) and UDP-glucuronosyltransferases (UGTs): evidence for the functionally active UGT in P450-UGT complex. *Drug Metab. Pharmacokinet.* 22: 367–376. 17965520 10.2133/dmpk.22.367

[R45] Itkin M. (2013) Biosynthesis of antinutritional. *Science* 341: 175–179. 23788733 10.1126/science.1240230

[R46] Iwamoto M., Horikawa C., Shikata M., Wasaka N., Kato T. and Sato H. (2014) Stinging hairs on the Japanese nettle Urtica thunbergiana have a defensive function against mammalian but not insect herbivores. *Ecol. Res.* 29: 455–462.

[R47] Jensen N.B., Zagrobelny M., Hjernø K., Olsen C.E., Houghton-Larsen J., Borch J., et al. (2011) Convergent evolution in biosynthesis of cyanogenic defence compounds in plants and insects. *Nat. Commun.* 2: 273. 10.1038/ncomms1271 PMC4354137 21505429

[R48] Jones J.D.G., Vance R.E. and Dangl J.L. (2016) Intracellular innate immune surveillance devices in plants and animals. *Science* 54: aaf6395. 10.1126/science.aaf6395 27934708

[R49] Kannangara R., Motawia M.S., Hansen N.K.K., Paquette S.M., Olsen C.E., Møller B.L., et al. (2011) Characterization and expression profile of two UDP-glucosyltransferases, UGT85K4 and UGT85K5, catalyzing the last step in cyanogenic glucoside biosynthesis in cassava. *Plant J.* 68: 287–301. 21736650 10.1111/j.1365-313X.2011.04695.x

[R50] Kawai Y., Ono E. and Mizutani M. (2014) Evolution and diversity of the 2-oxoglutarate-dependent dioxygenase superfamily in plants. *Plant J.* 78: 328–343. 24547750 10.1111/tpj.12479

[R51] Kazachkova Y., Zemach I., Panda S., Bocobza S., Vainer A., Rogachev I., et al. (2021) The GORKY glycoalkaloid transporter is indispensable for preventing tomato bitterness. *Nat. Plants* 7: 468–480. 33707737 10.1038/s41477-021-00865-6

[R52] Khersonsky O. and Tawfik D.S. (2010) Enzyme promiscuity: a methanistic and evolutionary perspective. *Annu. Rev. Biochem.* 79: 471–505. 20235827 10.1146/annurev-biochem-030409-143718

[R53] Kim J.-M., To T.K., Matsui A., Tanoi K., Kobayashi N.I., Matsuda F., et al. (2017) Acetate-mediated novel survival strategy against drought in plants. *Nat. Plants* 3: 17097. 10.1038/nplants.2017.97 28650429

[R54] Kirsch R., Okamura Y., Haeger W., Vogel H., Kunert G. and Pauchet Y. (2022) Metabolic novelty originating from horizontal gene transfer is essential for leaf beetle survival. *Proc. Natl. Acad. Sci. USA* 119: e2205857119. 10.1073/pnas.2205857119 PMC9546569 36161953

[R55] Kumano T., Fujiki E., Hashimoto Y. and Kobayashi M. (2016) Discovery of a sesamin-metabolizing microorganism and a new enzyme. *Proc. Natl. Acad. Sci. USA* 113: 9087–9092. 27444012 10.1073/pnas.1605050113 PMC4987775

[R56] Kumar S., Suleski M., Craig J.M., Kasprowicz A.E., Sanderford M., et al. (2022) TimeTree 5: an expanded resource for species divergence times. *Mol. Biol. Evol.* 39: msac174. 10.1093/molbev/msac174 PMC9400175 35932227

[R57] Kuzmin E., Vandersluis B., Ba A.N.N., Wang W., Koch E.N., Usaj M., et al. (2020) Exploring whole-genome duplicate gene retention with complex genetic interaction analysis. *Science* 368: eaaz5667. 10.1126/science.aaz5667 PMC7539174 32586993

[R58] Lanier E.R., Andersen T.B. and Hamberger B. (2023) Plant terpene specialized metabolism: complex networks or simple linear pathways? *Plant J.* 114: 1178–1201. 36891828 10.1111/tpj.16177 PMC11166267

[R59] La Peña R D., Hodgson H., Liu J.C., Stephenson M.J., Martin A.C., et al. (2023) Complex scaffold remodeling in plant triterpene biosynthesis. *Science* 379: 361–368. 36701471 10.1126/science.adf1017 PMC9976607

[R60] Laursen T., Borch J., Knudsen C., Bavishi K., Torta F., Martens H.J., et al. (2016) Characterization of a dynamic metabolon producing the defense compound dhurrin in sorghum. *Science* 354: 890–893. 27856908 10.1126/science.aag2347

[R61] Lau W. and Sattely E.S. (2015) Six enzymes from mayapple that complete the biosynthetic pathway to the etoposide aglycone. *Science* 349: 1224–1228. 26359402 10.1126/science.aac7202 PMC6861171

[R62] Liang M., Chen W., LaFountain A.M., Liu Y., Peng F., Xia R., et al. (2023) Taxon-specific, phased siRNAs underlie a speciation locus in monkeyflowers. *Science* 379: 576–582. 36758083 10.1126/science.adf1323 PMC10601778

[R63] Li J., Halitschke R., Li D., Paetz C., Su H., Heiling S., et al. (2021) Controlled hydroxylations of diterpenoids allow for plant chemical defense without autotoxicity. *Science* 371: 255–260. 33446550 10.1126/science.abe4713

[R64] Li Y., Winzer T., He Z. and Graham I.A. (2020) Over 100 million years of enzyme evolution underpinning the production of morphine in the Papaveraceae family of flowering plants. *Plant Commun.* 1: 100029. 10.1016/j.xplc.2020.100029 PMC7357826 32685922

[R65] Lou Y.R., Anthony T.M., Fiesel P.D., Arking R.E., Christensen E.M., Jones A.D., et al. (2021) It happened again: convergent evolution of acylglucose specialized metabolism in black nightshade and wild tomato. *Sci. Adv.* 7: eabj8726. 10.1126/sciadv.abj8726 PMC8580325 34757799

[R66] Lüthi M.N., Berardi A.E., Mandel T., Freitas L.B. and Kuhlemeier C. (2022) Single gene mutation in a plant MYB transcription factor causes a major shift in pollinator preference. *Curr. Biol.* 32: 5295–5308. 36473466 10.1016/j.cub.2022.11.006

[R67] Mao L., Kawaide H., Higuchi T., Chen M., Miyamoto K., Hirata Y., et al. (2020) Genomic evidence for convergent evolution of gene clusters for momilactone biosynthesis in land plants. *Proc. Natl. Acad. Sci. USA* 117: 12472–12480. 32409606 10.1073/pnas.1914373117 PMC7275736

[R68] Martens S. and Mithöfer A. (2005) Flavones and flavone synthases. *Phytochemistry* 66: 2399–2407. 16137727 10.1016/j.phytochem.2005.07.013

[R69] Matsuno M., Compagnon V., Schoch G.A., Schmitt M., Debayle D., Bassard J.-E., et al. (2009) Evolution of a novel phenolic pathway for pollen development. *Science* 325: 1688–1692. 19779199 10.1126/science.1174095

[R70] Molitor C., Mauracher S.G. and Rompel A. (2016) Aurone synthase is a catechol oxidase with hydroxylase activity and provides insights into the mechanism of plant polyphenol oxidases. *Proc. Natl. Acad. Sci. USA* 113: E1806–E1815. 26976571 10.1073/pnas.1523575113 PMC4822611

[R71] Mori S., Hasegawa Y. and Moriguchi Y. (2023) Color strategies of camellias recruiting different pollinators. *Phytochemistry* 207: 113559. 10.1016/j.phytochem.2022.113559 36528119

[R72] Munakata R., Olry A., Takemura T., Tatsumi K., Ichino T., Villard C., et al. (2021) Parallel evolution of UbiA superfamily proteins into aromatic *O*-prenyltransferases in plants. *Proc. Natl. Acad. Sci. USA* 118: e2022294118. 10.1073/pnas.2022294118 PMC8092402 33883279

[R73] Muralidharan M., Buss K., Larrimore K.E., Segerson N.A., Kannan L. and Mor T.S. (2013) The *Arabidopsis thaliana* ortholog of a purported maize cholinesterase gene encodes a GDSL-lipase. *Plant Mol. Biol.* 81: 565–576. 23430565 10.1007/s11103-013-0021-8 PMC3769184

[R74] Murata J., Watanabe T. and Komura H. (2022) Bacterial volatile isovaleric acid triggers growth alteration of Arabidopsis seedlings. *Metabolites* 12: 1043. 10.3390/metabo12111043 PMC9699611 36355126

[R75] Murata J., Watanabe T., Sugahara K., Yamagaki T. and Takahashi T. (2015) High-resolution mass spectrometry for detecting acetylcholine in Arabidopsis. *Plant Signal Behav.* 10: e1074367. 10.1080/15592324.2015.1074367 PMC4883864 26237653

[R76] Nakayama T., Takahashi S. and Waki T. (2019) Formation of flavonoid metabolons: functional significance of protein-protein interactions and impact on flavonoid chemodiversity. *Front. Plant Sci.* 10: 821. 10.3389/fpls.2019.00821 PMC6629762 31338097

[R77] Nakayama T., Yonekura-Sakakibara K., Sato T., Kikuchi S., Fukui Y., Fukuchi-Mizutani M., et al. (2000) Aureusidin synthase: a polyphenol oxidase homolog responsible for flower coloration. *Science* 290: 1163–1166. 11073455 10.1126/science.290.5494.1163

[R78] Nakayasu M., Takamatsu K., Yazaki K. and Sugiyama A. (2022) Plant specialized metabolites in the rhizosphere of tomatoes: secretion and effects on microorganisms. *Biosci. Biotechnol. Biochem.* 87: 13–20. 36373409 10.1093/bbb/zbac181

[R79] Negri S., Commisso M., Avesani L. and Guzzo F. (2021) The case of tryptamine and serotonin in plants: a mysterious precursor for an illustrious metabolite. *J. Exp. Bot.* 72: 5336–5355. 34009335 10.1093/jxb/erab220

[R80] Nelson D. and Werck-Reichhart D. (2011) A P450-centric view of plant evolution. *Plant J*. 66: 194–211. 21443632 10.1111/j.1365-313X.2011.04529.x

[R81] Nitsch S., Zorro Shahidian L. and Schneider R. (2021) Histone acylations and chromatin dynamics: concepts, challenges, and links to metabolism. *EMBO Rep.* 22: 1–13. 10.15252/embr.202152774 PMC8406397 34159701

[R82] Noguchi A., Horikawa M., Murata J., Tera M., Kawai Y., Ishiguro M., et al. (2014) Mode-of-action and evolution of methylenedioxy bridge forming P450s in plant specialized metabolism. *Plant Biotechnol.* 31: 493–503.

[R83] Obayashi T., Hibara H., Kagaya Y., Aoki Y. and Kinoshita K. (2022) ATTED-II v11: a plant gene coexpression database using a sample balancing technique by subagging of principal components. *Plant Cell Physiol.* 63: 869–881. 35353884 10.1093/pcp/pcac041

[R84] O’donnell A.J., Huang R., Barboline J.J. and Barkman T.J. (2021) Convergent biochemical pathways for xanthine alkaloid production in plants evolved from ancestral enzymes with different catalytic properties. *Mol. Biol. Evol.* 38: 2704–2714. 33662138 10.1093/molbev/msab059 PMC8233510

[R85] Ogino A., Taniguchi F., Yoshida K., Matsumoto S., Fukuoka H. and Nesumi A. (2019) A new DNA marker cafless-TCS1 for selection of caffeine-less tea plants. *Breed. Sci.* 69: 393–400. 31598071 10.1270/jsbbs.18161 PMC6776138

[R86] Ohgami S., Ono E., Horikawa M., Murata J., Totsuka K., Toyonaga H., et al. (2015) Volatile glycosylation in tea plants: sequential glycosylations for the biosynthesis of aroma β-primeverosides are catalyzed by two *Camellia sinensis* glycosyltransferases. *Plant Physiol.* 168: 464–477. 25922059 10.1104/pp.15.00403 PMC4453793

[R87] Ono E., Nakai M., Fukui Y., Tomimori N., Fukuchi-Mizutani M., Saito M., et al. (2006) Formation of two methylenedioxy bridges by a *Sesamum* CYP81Q protein yielding a furofuran lignan, (+)-sesamin. *Proc. Natl. Acad. Sci. USA* 103: 10116–10121. 16785429 10.1073/pnas.0603865103 PMC1502515

[R88] Ono E., Waki T., Oikawa D., Murata J., Shiraishi A., Toyonaga H., et al. (2020) Glycoside-specific glycosyltransferases catalyze regio-selective sequential glucosylations for a sesame lignan, sesaminol triglucoside. *Plant J.* 101: 1221–1233. 31654577 10.1111/tpj.14586

[R89] Pandey A.V., Henderson C.J., Ishii Y., Kranendonk M., Backes W.L. and Zanger U.M. (2017) Editorial: role of protein-protein interactions in metabolism: genetics, structure, function. *Front. Pharmacol.* 8: 8–10. 29230176 10.3389/fphar.2017.00881 PMC5712015

[R90] Pareek V., Sha Z., He J., Wingreen N.S. and Benkovic S.J. (2021) Metabolic channeling: predictions, deductions, and evidence. *Mol. Cell* 81: 3775–3785. 34547238 10.1016/j.molcel.2021.08.030 PMC8485759

[R91] Qin C., Ahanger M.A., Lin B., Huang Z., Zhou J., Ahmed N., et al. (2021) Comparative transcriptome analysis reveals the regulatory effects of acetylcholine on salt tolerance of *Nicotiana benthamiana*. *Phytochemistry* 181: 112582. 10.1016/j.phytochem.2020.112582 33246307

[R92] Qu Y., Safonova O. and De Luca V. (2019) Completion of the canonical pathway for assembly of anticancer drugs vincristine/vinblastine in Catharanthus roseus. *Plant J.* 97: 257–266. 30256480 10.1111/tpj.14111

[R93] Rausher M.D., Miller R.E. and Tiffin P. (1999) Patterns of evolutionary rate variation among genes of the anthocyanin biosynthetic pathway. *Mol. Biol. Evol.* 16: 266–274. 10028292 10.1093/oxfordjournals.molbev.a026108

[R94] Roy R., Moreno N., Brockman S.A., Kostanecki A., Zambre A., Holl C., et al. (2022) Convergent evolution of a blood-red nectar pigment in vertebrate-pollinated flowers. *Proc. Natl. Acad. Sci. USA* 119: e2114420119. 10.1073/pnas.2114420119 PMC8812537 35074876

[R95] Sagane Y., Nakagawa T., Yamamoto K., Michikawa S., Oguri S. and Momonoki Y.S. (2005) Molecular characterization of maize acetylcholinesterase: a novel enzyme family in the plant kingdom. *Plant Physiol.* 138: 1359–1371. 15980188 10.1104/pp.105.062927 PMC1176409

[R96] Sánchez-Pérez R., Pavan S., Mazzeo R., Moldovan C., Aiese Cigliano R., Del Cueto J., et al. (2019) Mutation of a bHLH transcription factor allowed almond domestication. *Science* 364: 1095–1098. 31197015 10.1126/science.aav8197

[R97] Sayama T., Ono E., Takagi K., Takada Y., Horikawa M., Nakamoto Y., et al. (2012) The *Sg-1* glycosyltransferase locus regulates structural diversity of triterpenoid saponins of soybean. *Plant Cell* 24: 2123–2138. 22611180 10.1105/tpc.111.095174 PMC3442591

[R98] Schmitt D.L. and An S. (2017) Spatial organization of metabolic enzyme complexes in cells. *Biochemistry* 56: 3184–3196. 28580779 10.1021/acs.biochem.7b00249 PMC5574030

[R99] Shang Y., Ma Y., Zhou Y., Zhang H., Duan L., Chen H., et al. (2014) Biosynthesis, regulation, and domestication of bitterness in cucumber. *Science* 346: 1084–1088. 25430763 10.1126/science.1259215

[R100] Shoji T. (2019) The recruitment model of metabolic evolution: jasmonate-responsive transcription factors and a conceptual model for the evolution of metabolic pathways. *Front. Plant Sci.* 10: 560. 10.3389/fpls.2019.00560 PMC6528166 31156658

[R101] Sirikantaramas S., Yamazaki M. and Saito K. (2008) Mutations in topoisomerase I as a self-resistance mechanism coevolved with the production of the anticancer alkaloid camptothecin in plants. *Proc. Natl. Acad. Sci. USA* 105: 6782–6786. 18443285 10.1073/pnas.0801038105 PMC2365566

[R102] Stringlis I.A., De Jonge R. and Pieterse C.M.J. (2019) The age of coumarins in plant-microbe interactions. *Plant Cell Physiol.* 60: 1405–1419. 31076771 10.1093/pcp/pcz076 PMC6915228

[R103] Sugahara K., Kitao K., Yamagaki T. and Koyama T. (2020) Practical optimization of liquid chromatography/mass spectrometry conditions and pretreatment methods toward the sensitive quantification of auxin in plants. *Rapid Commun. Mass Spectrom.* 34: e8625. 10.1002/rcm.8625 31658390

[R104] Summers R.M., Mohanty S.K., Gopishetty S. and Subramanian M. (2015) Genetic characterization of caffeine degradation by bacteria and its potential applications. *Microb. Biotechnol.* 8: 369–378. 25678373 10.1111/1751-7915.12262 PMC4408171

[R105] Takos A.M., Knudsen C., Lai D., Kannangara R., Mikkelsen L., Motawia M.S., et al. (2011) Genomic clustering of cyanogenic glucoside biosynthetic genes aids their identification in *Lotus japonicu*s and suggests the repeated evolution of this chemical defence pathway. *Plant J.* 68: 273–286. 21707799 10.1111/j.1365-313X.2011.04685.x

[R106] Tanaka Y., Asano T., Kanemitsu Y., Goto T., Yoshida Y., Yasuba K., et al. (2019) Positional differences of intronic transposons in pAMT affect the pungency level in chili pepper through altered splicing efficiency. *Plant J.* 100: 693–705. 31323150 10.1111/tpj.14462

[R107] Tatsis E.C., Carqueijeiro I., Dugé de Bernonville T., Franke J., Dang T.-T.-T., Oudin A., et al. (2017) A three enzyme system to generate the Strychnos alkaloid scaffold from a central biosynthetic intermediate. *Nat. Commun.* 8: 316. 10.1038/s41467-017-00154-x PMC5566405 28827772

[R108] Thodberg S., Del Cueto J., Mazzeo R., Pavan S., Lotti C., Dicenta F., et al. (2018) Elucidation of the amygdalin pathway reveals the metabolic basis of bitter and sweet almonds (*Prunus dulcis*). *Plant Physiol.* 178: 1096–1111. 30297455 10.1104/pp.18.00922 PMC6236625

[R109] Tikunov Y.M., Molthoff J., de Vos R.C.H., Beekwilder J., van Houwelingen A., van der Hooft J.J.J., et al. (2013) Non-smoky GLYCOSYLTRANSFERASE1 prevents the release of smoky aroma from tomato fruit. *Plant Cell* 25: 3067–3078. 23956261 10.1105/tpc.113.114231 PMC3784599

[R110] Toyota M., Spencer D., Sawai-Toyota S., Jiaqi W., Zhang T., Koo A.J., et al. (2018) Glutamate triggers long-distance, calcium-based plant defense signaling. *Science* 361: 1112–1115. 30213912 10.1126/science.aat7744

[R111] Tran H.T., Nguyen G.T., Nguyen H.H.T., Tran H.T., Tran Q.H., Tran Q.H., et al. (2022) Isolation and cytotoxic potency of endophytic fungi associated with dysosma difformis, a study for the novel resources of podophyllotoxin. *Mycobiology* 50: 389–398. 36404896 10.1080/12298093.2022.2126166 PMC9645267

[R112] Tudzynski B. (2005) Gibberellin biosynthesis in fungi: genes, enzymes, evolution, and impact on biotechnology. *Appl. Microbiol. Biotechnol.* 66: 597–611. 15578178 10.1007/s00253-004-1805-1

[R113] Ukken F.P., Dowell N.L., Hajra M. and Carroll S.B. (2022) A novel broad spectrum venom metalloproteinase autoinhibitor in the rattlesnake Crotalus atrox evolved via a shift in paralog function. *Proc. Natl. Acad. Sci. USA* 119: e2214880119. 10.1073/pnas.2214880119 PMC9907073 36508672

[R114] Villard C., Munakata R., Kitajima S., van Velzen R., Schranz M.E., Larbat R., et al. (2021) A new P450 involved in the furanocoumarin pathway underlies a recent case of convergent evolution. *New Phytol.* 231: 1923–1939. 33978969 10.1111/nph.17458

[R115] Waki T., Mameda R., Nakano T., Yamada S., Terashita M., Ito K., et al. (2020) A conserved strategy of chalcone isomerase-like protein to rectify promiscuous chalcone synthase specificity. *Nat. Commun.* 11: 1–14. 32054839 10.1038/s41467-020-14558-9 PMC7018950

[R116] Weng J.K. (2014) The evolutionary paths towards complexity: a metabolic perspective. *New Phytol.* 201: 1141–1149. 23889087 10.1111/nph.12416

[R117] Weng J.K., Lynch J.H., Matos J.O. and Dudareva N. (2021) Adaptive mechanisms of plant specialized metabolism connecting chemistry to function. *Nat. Chem. Biol.* 17: 1037–1045. 34552220 10.1038/s41589-021-00822-6

[R118] Wilson A.E. and Tian L. (2019) Phylogenomic analysis of UDP-dependent glycosyltransferases provides insights into the evolutionary landscape of glycosylation in plant metabolism. *Plant J.* 100: 1273–1288. 31446648 10.1111/tpj.14514

[R119] Yamamura M., Kumatani M., Shiraishi A., Matsuura Y., Kobayashi K., Suzuki A., et al. (2023) Two *O*-methyltransferases from phylogenetically unrelated cow parsley (*Anthriscus sylvestris*) and hinoki-asunaro (*Thujopsis dolabrat*a var. hondae) as a signature of lineage-specific evolution in lignan biosynthesis. *Plant Cell Physiol.* 64: 124–147. 36412832 10.1093/pcp/pcac164

[R120] Yamazaki Y., Sudo H., Yamazaki M., Aimi N. and Saito K. (2003) Camptothecin biosynthetic genes in hairy roots of *Ophiorrhiza pumila*: cloning, characterization and differential expression in tissues and by stress compounds. *Plant Cell Physiol.* 44: 395–403. 12721380 10.1093/pcp/pcg051

[R121] Yang M., Fehl C., Lees K.V., Lim E.K., Offen W.A., Davies G.J., et al. (2018) Functional and informatics analysis enables glycosyltransferase activity prediction. *Nat. Chem. Biol.* 14: 1109–1117. 30420693 10.1038/s41589-018-0154-9

[R122] Zhang Y., Beard K.F.M., Swart C., Bergmann S., Krahnert I., Nikoloski Z., et al. (2017) Protein-protein interactions and metabolite channelling in the plant tricarboxylic acid cycle. *Nat. Commun.* 8: 15212. 10.1038/ncomms15212 PMC5440813 28508886

[R123] Zhang Y. and Fernie A.R. (2021) Metabolons, enzyme–enzyme assemblies that mediate substrate channeling, and their roles in plant metabolism. *Plant Commun.* 2: 100081. 10.1016/j.xplc.2020.100081 PMC7816073 33511342

[R124] Zhan C., Shen S., Yang C., Liu Z., Fernie A.R., Graham I.A., et al. (2022) Plant metabolic gene clusters in the multi-omics era. *Trends Plant Sci.* 27: 981–1001. 35365433 10.1016/j.tplants.2022.03.002

[R125] Zhao E.M., Suek N., Wilson M.Z., Dine E., Pannucci N.L., Gitai Z., et al. (2019) Light-based control of metabolic flux through assembly of synthetic organelles. *Nat. Chem. Biol.* 15: 589–597. 31086330 10.1038/s41589-019-0284-8 PMC6755918

[R126] Zhao X., Zhao Y., Gou M. and Liu C. (2023) Tissue-preferential recruitment of electron transfer chains for cytochrome P450-catalyzed phenolic biosynthesis. *Sci. Adv.* 9: eade4389. 10.1126/sciadv.ade4389 PMC9833660 36630494

[R127] Zheng L., Li C., Ma X., Zhou H., Liu Y., Wang P., et al. (2021) Functional interplay of histone lysine 2-hydroxyisobutyrylation and acetylation in Arabidopsis under dark-induced starvation. *Nucleic Acids Res.* 49: 7347–7360. 34165567 10.1093/nar/gkab536 PMC8287917

[R128] Zhong Y., Xun W., Wang X., Tian S., Zhang Y., Li D., et al. (2022) Root-secreted bitter triterpene modulates the rhizosphere microbiota to improve plant fitness. *Nat. Plants* 8: 887–896. 35915145 10.1038/s41477-022-01201-2

[R129] Zhou Y., Ma Y., Zeng J., Duan L., Xue X., Wang H., et al. (2016) Convergence and divergence of bitterness biosynthesis and regulation in Cucurbitaceae. *Nat. Plants* 2: 1–8. 10.1038/nplants.2016.183 PMC5449191 27892922

