# Supplementary material for: Exploring the Evolvability of Plant Specialized Metabolism: Uniqueness Out Of Uniformity and Uniqueness Behind Uniformity
Source: Plant Cell Physiol. 2023 Jun 12;64(12):1449–65. doi: 10.1093/pcp/pcad057 (PMC10734894; doi:10.1093/pcp/pcad057)
Supplement: pcad057_Supp [file pcad057_supp.zip › suppl_data/pcp-2023-e-00052-File007.pdf]

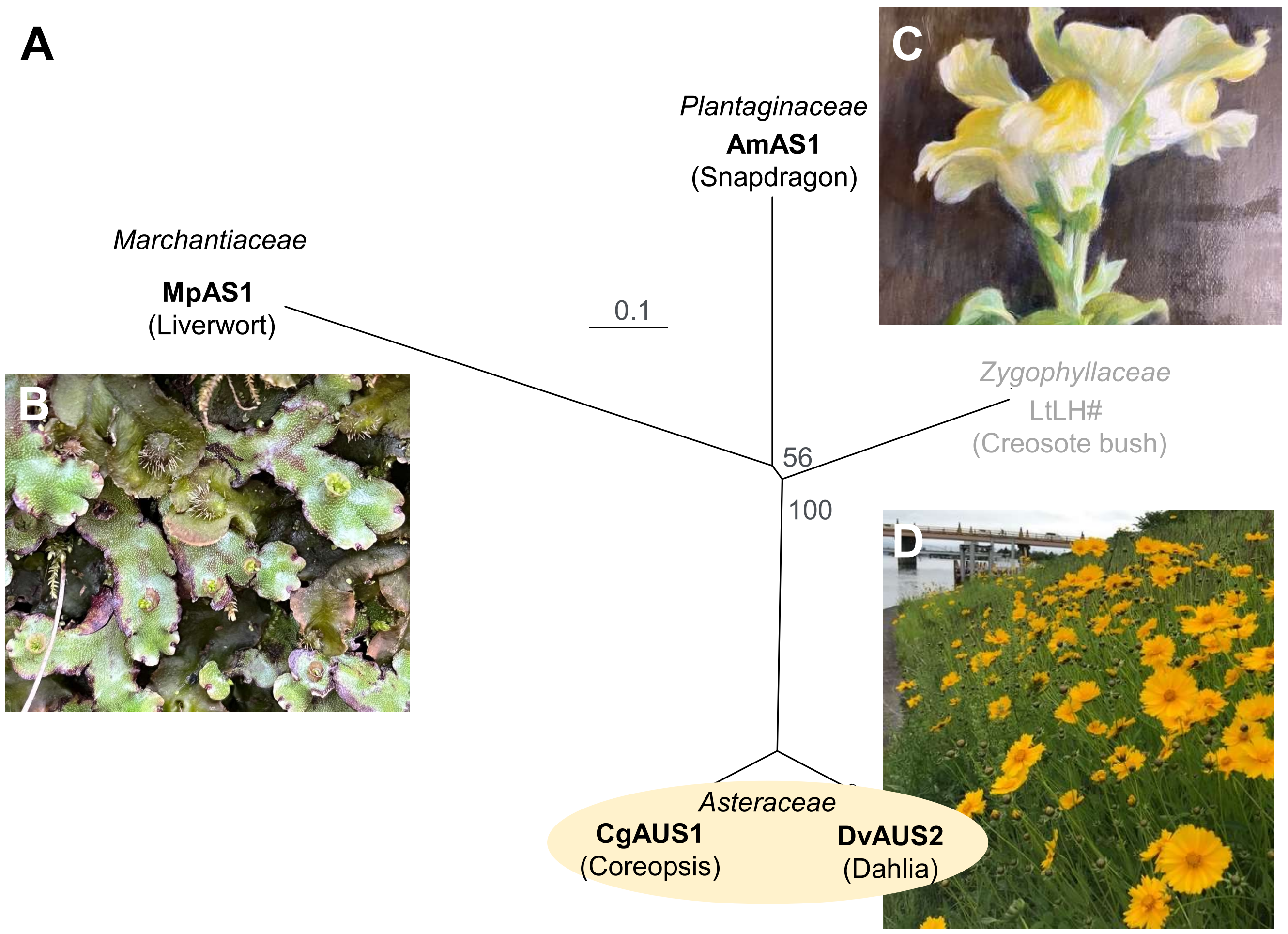

E

Family	Marchantiaceae	Plantaginaceae	Zygophyllaceae	Asteraceae	Asteraceae
Name	Liverwort	Snapdragon	Cresote bush	Coreopsis	Dahlia
PPO	MpAS1	AmAS1	LtLH#	CgAUS1	DvAUS2
Accession	Mapoly0021s0041	AB044884	AY370019	KC878308	MT181750
Mp	100				
Am	24	100			
Lt	21	42	100		
Cg	19	33	39	100	
Dv	18	35	39	78	100

Supplementary Figure S1. Phylogenetic relationships among aurone biosynthetic PPOs
(A) A Neighbor-Joining phylogenetic tree of aurone synthetic PPOs; Snapdragon AmAS1 (Nakayama et al., 2000), Coreopsis CgAUS1 (Molitor et al., 2016), DvAUS2, and liverwort MpAS1 (Berland et al., 2019, Furudate et al., 2023) with a specialized lignan synthetic PPO, larreatricin 3-hydroxylase (LtLH) of the creosote bush (Cho et al., 2003). The scale bar indicates 0.1 amino acid substitutions per site. Bootstrap values are indicated on the branches (1000 replicates). (B) A photo of Marchantia spp. grown near the Sanda river, Otsu, Shiga prefecture, Japan. (C) A painting of yellow snapdragon flowers by Shinichi Asano. (D) A photo of Coreopsis flowers from Seta Karahashi, Otsu, Shiga prefecture, Japan. (E) Pairwise amino acid identity (%) of aurone biosynthetic PPOs with a non-aurone biosynthetic LtLH indicated by #. High sequence similarity (78%) between Coreopsis AUS1 and Dahlia AUS2 suggests the common origin of aurone biosynthetic PPO in the Asteraceae lineage.
